# Anatomical Aspects and Long-Term Outcomes of Additional Surgical Repair During Heart Transplantation in Adult Congenital Heart Disease

**DOI:** 10.1097/MAT.0000000000002353

**Published:** 2024-11-25

**Authors:** Nicola Pradegan, Claudia Cattapan, Chiara Tessari, Giuseppe Toscano, Augusto D’Onofrio, Vincenzo Tarzia, Antonio Gambino, Marny Fedrigo, Vladimiro L. Vida, Annalisa Angelini, Gino Gerosa

**Affiliations:** From the * Cardiac Surgery Unit, Cardio-Thoraco-Vascular and Public Health Department, Padova University Hospital, Padova, Italy; † Cardiovascular Pathology, Cardio-Thoraco-Vascular and Public Health Department, Padova University Hospital, Padova, Italy; ‡ Pediatric Cardiac Surgery Unit, Cardio-Thoraco-Vascular and Public Health Department, Padova University Hospital, Padova, Italy.

**Keywords:** adult congenital heart disease, heart transplantation, complex congenital heart disease

## Abstract

Adult patients with congenital heart disease (ACHD) requiring heart transplantation (HT) usually show complex anatomies, posing surgical challenges. Consequently, we analyzed technical aspects and early and long-term outcomes of additional surgical repairs during HT in ACHD. Forty patients were identified (23 males, median age: 38 years, interquartile range [IQR]: 26–50). Of these, 17 (42.5%) required additional surgical repair (7 systemic veins repair, 13 pulmonary arteries repair). These procedures were more associated with univentricular physiology (*p* < 0.001) and prior Fontan palliation (*p* < 0.001). Eight (20.0%) experienced 30 day mortality. At a median follow-up of 5.6 (IQR: 2.0–11.9) years, 5 (12.5%) patients died. Additional surgical repair did not affect postoperative 30 day and long-term follow-up mortality (*p* = 0.451 and *p* = 0.330, respectively).

## Background

Advancements in surgical techniques and postoperative care have led to a significant increase in the number of children who underwent congenital heart disease (CHD) surgery reaching adulthood.^1^ Unfortunately, many of these adult CHD (ACHD) patients experience worsening heart failure during over time.^2^ Although ACHD patients represent a small portion (around 3%) of heart transplant (HT) recipients, their numbers are steadily rising (40% in the last 20 years).^2^ Most CHDs involve abnormalities in the pulmonary artery, aorta, or systemic veins which significantly complicate HT surgery.^3^

Unfortunately, a critical knowledge gap exists regarding the optimal surgical management of these anatomical variations during HT and their early and long-term outcomes.

## Aims

This study aimed to review the anatomical characteristics of ACHD undergoing HT, the need for additional surgical repairs during HT, and their effect on early and late outcomes.

## Materials and Methods

The current study was approved by our local Ethics Committee (EC) (clinical registration number: 343n/AO/23). Written patient informed consent was waived by our EC.

**Figure 1.**
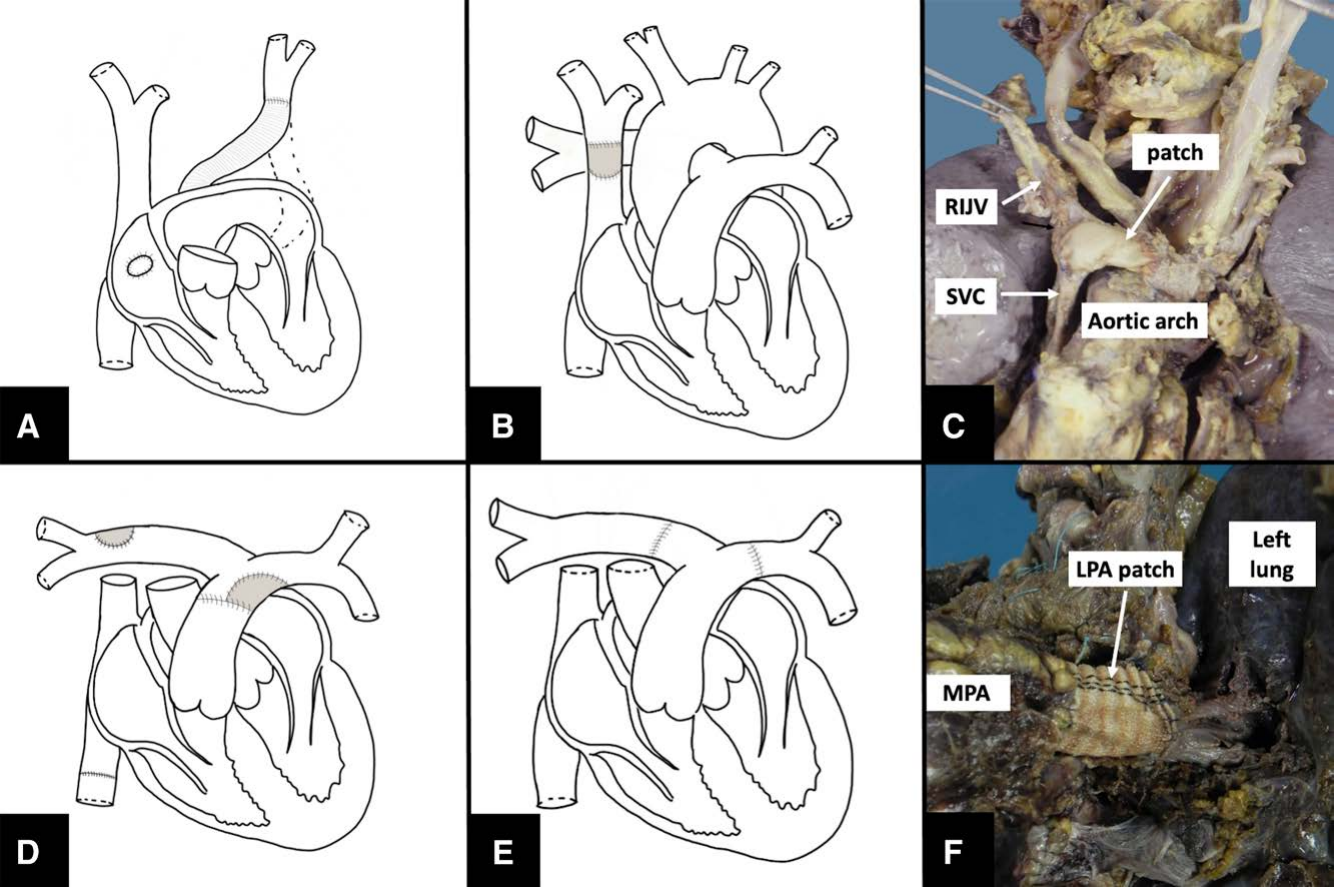
Pictures representing systemic veins and PA defects repair at the time of heart transplantation. **A**: Re-rooting of LSVC to right atrium by means of an artificial conduit. **B**: SVC elongation with a conduit. **C**: Postmortem pathological specimen photo showing anonymous vein-SVC enlargement pericardial patch (RIJV). **D**: Pulmonary arteries repair with a patch and IVC elongation with a prosthesis. **E**: Bifurcated donor PA. **F**: Postmortem pathological specimen picture showing the LPA dacron patch enlargement (MPA). IVC, inferior vena cava; LPA, left pulmonary artery; LSVC, left superior vena cava; MPA, main pulmonary artery; PA, pulmonary artery; RIJV, right internal jugular vein; SVC, superior vena cava.

Data on all ACHD patients greater than or equal to 16 years of age who underwent HT at our center between November 1985 and June 2023 were retrospectively collected. Surgical charts were reviewed to assess surgical anatomy and any additional repair required during HT. Intraoperative systemic veins repair was defined as: left superior vena cava (LSVC) connection to the right atrium with a conduit (Figure 1A), superior (Figure 1, B and C) or inferior vena cava (Figure 1D) (SVC or IVC, respectively) enlargement using a patch, or their elongation with a conduit. Intraoperative pulmonary artery repair was defined as: cases in which the main or left and right pulmonary arteries (MPA, LPA, RPA) were enlarged using pericardial patches (Figure 1, D–F), or elongated with artificial conduits or a bifurcated donor graft (Figure 1E). The primary endpoint was 30 day and follow-up mortality. Secondary endpoints included: freedom from post-HT surgical or interventional procedures to correct residual pulmonary or systemic vein abnormalities and freedom from follow-up right or left ventricle dysfunction, moderate-to-severe mitral or tricuspid valve regurgitation, and severe cardiac allograft vasculopathy (CAV). Both primary and secondary endpoints were compared between patients who underwent intraoperative repair and those who did not.

Continuous variables were summarized using median and interquartile range (IQR). Categorical variables were summarized by frequency and percentage. Survival rates and freedom from follow-up complications were estimated using Kaplan–Meier method. A comparison analysis between patients requiring an intraoperative additional repair or not was performed using the Mann–Whitney *U* test (for continuous variables) or Pearson’s chi-square test (for categorical variables). All analyses were performed using SPSS version 28.0.1.0 (SPSS Statistics; IBM, NY). *p* Values less than 0.05 were considered statistically significant.

## Results

Among all those patients who underwent HT during the study period, we identified 40 ACHD patients. Twenty-three (57.5%) of these patients underwent HT in the last decade (2013–2023). Notably, 12 (30.0%) of these patients had univentricular physiology (9 of whom had prior Fontan surgery). Previous cardiac surgeries were performed in 35 (87.5%) patients.

Seventeen (42.5%) patients required additional surgical repairs at the time of HT: 7 on systemic veins and 13 on pulmonary arteries. No patients required additional aortic procedures.

**Figure 2.**
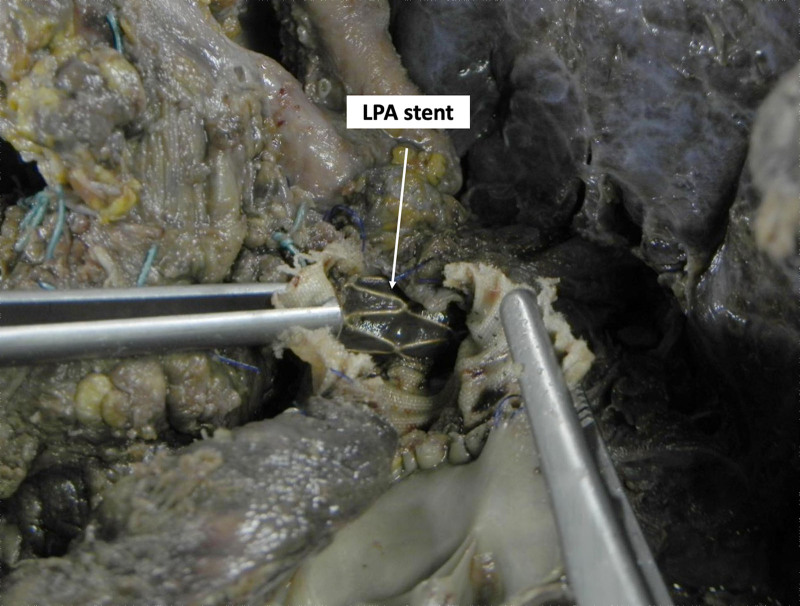
Postmortem pathological photo showing the LPA stent within a reconstructed LPA (LPA elongation with a dacron conduit) at the time of heart transplantation. LPA, left pulmonary artery.

Thirty-day mortality occurred in 8 (20.0%) patients and none of these deaths were related to surgical complications at the time of HT. At a median follow-up of 5.6 (IQR: 2.0–11.9) years, 5 (19.2%) patients died. These patients belonged to the first and second decade of the HT program, and only three patients died because of cardiac-related causes. Only 1 patient required an additional percutaneous procedure after HT: a 17 year old patient, with a history of hypoplastic left heart syndrome who underwent a Fontan operation and who required an MPA reconstruction with an artificial conduit; unfortunately, 13 days after the HT, he required LPA stenting (Figure 2), but died 3 days after this procedure due to lung complications. No patients required additional postoperative surgical or interventional procedures during follow-up. Table 1, Supplemental Digital Content, http://links.lww.com/ASAIO/B370, summarizes patient characteristics in the overall cohort, and among those who underwent additional repair or not.

Compared to recipients who did not require additional repairs during HT, those who did were more likely to have univentricular physiology (*p* < 0.001) and a prior Fontan procedure (*p* < 0.001). No difference was found in terms of postoperative need of circulatory mechanical support between the two subgroups (additional repair group: n = 6[35.3%] *versus* no repair group: n = 5[21.7%], *p* = 0.304); 30 day (additional repair group: n = 5[29.4%] *versus* no repair group: n = 3[13.0%]) and follow-up mortality (additional repair group: n = 0 *versus* no repair group: n = 5[21.7%]) also did not differ among those two groups (*p* = 0.451 and *p* = 0.330, respectively). Unfortunately, follow-up time in the additional repair cohort was shorter (*p* = 0.049). Similarly, no significant differences were found in terms of severe CAV (*p* = 0.086), biventricular function (left ejection fraction *p* = 0.0495, right shortening fraction *p* = 0.397), or moderate-to-severe atrioventricular valve regurgitation (tricuspid *p* = 0.513, mitral *p* = 0.308) during follow-up.

## Discussion

Our analysis shows that additional cardiac repair at the time of HT in ACHD is frequently required (~50% of our patients). Indeed, HT in ACHD often follows previous palliations (*eg*, Fontan operation, systemic-to-pulmonary shunts, PA banding) or surgical repairs (*eg*, valve repair/replacement, septal repair).^4–6^ Most of these past surgeries involve PA or systemic veins. Pulmonary artery repairs during HT might be required by accidental tears during surgical reentry, needing patch enlargements. For complex cases with abnormal PA growth, patch reconstruction can be challenging. In such situations, utilizing a donor heart with a bifurcated PA (if available) offers a valuable solution.^7^ Another significant challenge arises when abnormal vein or artery positioning is inherent to the CHD (*eg*, LSVC with absent innominate vein, situs inversus, dextrocardia).^8^ Connecting the donor heart to these abnormal structures may necessitate vessel elongation with patches (biological or artificial) or even tubular prostheses. Even if caval anastomotic stenosis is not an infrequent HT complication and might predispose to blood stasis and thrombus formation, abnormal venous rerouting during ACHD-HT might increase this risk. Unfortunately, our group is too limited to make any conclusions regarding this issue. As reported in other diseases and surgeries,^9^ a flow radiological analysis after systemic vein repair in ACHD-HT might help in understanding the rheological risk of this strategy.

Even if ACHD-HT is associated with reported high operative mortality (17.4% of 30 day death in a meta-analysis by Doumouras *et al*.; 16–19% among different ACHD groups in a large multicenter study by Davies *et al*.),^10,11^ our study found no statistically significant difference in early or late outcomes between patients requiring additional repairs and those who did not. However, absolute numbers disfavor additional repair patients who are preferentially univentricular, as already seen by Dib *et al*.^12^ Indeed, additional repair was more frequently performed in the last decade. This could be attributed to several factors: 1) surgeries performed by senior transplant surgeons with expertise in CHD, often collaborating with congenital heart surgeons, as recommended by current guidelines^13^; 2) advancement in surgical planning (*eg*, three-dimensional [3D] imaging reconstruction, 3D printing, minimally invasive procedures) to plan the best way to repair residual lesions before HT, potentially reducing surgical times.^14^

This study presents few limitations: first, it is a retrospective analysis at a single center with a small sample size. A larger, multicenter study would provide more statistically robust data. Additionally, the limited sample size precluded further subgroup analyses. Besides, much of the data regarding the immunological condition of the recipients before and after HT were missing, and this might justify our outcomes: despite a heterogenous distribution of PRA percentage and the type of surgeries before HT, we do not know if the previous operations (and the materials used), the pretransplant sensitization status, and the type of additional transplant repair might have influenced early and long-term overall outcomes as well as the rejection history of recipients.

In conclusion, our experience suggests that a significant proportion of ACHD patients undergoing HT require additional repairs of pulmonary arteries or systemic veins. These patients, even if more frequently associated with univentricular physiology, demonstrate similar short- and long-term outcomes compared to those without additional repairs.

## Supplementary Material
