# Supplementary material for: Anatomical Aspects and Long-Term Outcomes of Additional Surgical Repair During Heart Transplantation in Adult Congenital Heart Disease
Source: ASAIO J. 2024 Nov 25;71(7):e107–9. doi: 10.1097/MAT.0000000000002353 (PMC12199797; doi:10.1097/MAT.0000000000002353)
Supplement: Supplementary file 1 [file mat-71-e107-s001.pdf]

Supplementary file

Table 1. Pre- and post-transplant clinical characteristics.

**Pre- and post-operative variables**	**Total (n=40)**	**Additional repair (n=17)**	**No additional repair (n=23)**
Age (years)	38 (26-50)	36 (19-50)	39 (30-49)
Gender (male)	23 (57.5%)	10 (58.8%)	13 (56.5%)
Weight (kg)	62 (56-70)	62 (59-70)	53 (54-70)
Pre-transplant CHD			
- Ventricular septal defect	3 (7.5%)	1	2
- Partial atrio-ventricular canal	2 (5.0%)	-	2
- Complete atrio-ventricular canal	1 (2.5%)	1	-
- Tetralogy of Fallot	4 (10.0%)	1	3
- D-Transposition of Great Arteries	2 (5.0%)	-	2
- L- Transposition of Great Arteries	5 (12.5%)	3	2
- Ebstein malformation	4 (10.0%)	-	4
- Coarctation and bicuspid aortic valve	1 (2.5%)	-	1

- Valve dysplasia	4 (10.0%)	-	4
- Hypoplastic Left Heart Syndrome	2 (5.0%)	2	-
- Tricuspid atresia	2 (5.0%)	2	-
- Double Outlet Right Ventricle	2 (5.0%)	-	2
- Other	8 (20.0%)	7	1
Univentricular physiology	12 (30.0%)	11 (64.7%)	1 (4.3%)
Previous cardiac operations	35 (87.5%)	17 (100%)	18 (78.3%)
• Previous repair	21	6	15
• Previous palliation	15	12	3
◦ pulmonary artery banding	2	1	1
◦ systemic-to-pulmonary shunt	2	2	-
◦ Fontan	9	9	-
◦ Atrial switch	2	-	2
Number of reoperations:			
- 1	22	10	12
- 2	4	2	2

- > 2	8	5	3
Number of patients/decade<br>- <2004<br>- 2004-2013<br>- 2014-2023	 5<br>12<br>23	 -<br>3<br>14	 5<br>9<br>9
Left ventricle ejection fraction at the time of HT (%)	43 (25-59)	41 (30-51)	52 (25-64)
Pulmonary vascular resistances at the time of HT (WU/m^2)	1.5 (0.9-3.5)	1.7 (1.3-3.1)	3.1 (1.9-4.3)
Pre-transplant sensitization (Panel Reactive Antibody -PRA-):<br>- 0<br>- 1-50%<br>- >50%	 30 (75%)<br>6 (15%)<br>4 (10%)	 9<br>6<br>2	 21<br>-<br>2
Donor age (years)	40 (22-53)	40 (21-53)	39 (27-49)
Male donor	20 (50%)	7	13
Additional surgical repair at the time of HT<br>• Systemic veins repair	17 (42.5%)<br>7	 7	 -

• Pulmonary arteries repair	13	13	-
Cold ischemia time (minutes)	218 (166-262)	251 (195-279)	190 (145-237)
Cardiopulmonary bypass time (minutes)	333 (184-415)	399 (353-450)	200 (167-328)
Post-operative ECMO	10 (25.0%)	6 (35.3%)	4 (17.4%)
Post-operative Right Ventricular Assist Device	1 (2.5%)	-	1
Post-operative additional surgical or percutaneous procedures (except pericardial effusion drainage)	1	1	-
30-day mortality	8 (20%)	5 (29.4%)	3 (13.0%)
Follow-up time (years)	5.6 (2.0-11.9)	3.6 (1.6-5.3)	9.8 (4.0-13.4)
Cardiac Allograft Vasculopathy grade 2-3 (according to ISHLT guidelines) at follow-up	4 (13.8%)	-	4
Neoplasms at follow-up	1 (3.5%)	-	1
eGFR <60ml/min at follow-up	11 (37.9%)	2	9
TAPSE (mm) at follow-up	17 (IQR 13-19)	17 (14-19)	17 (13-19)

Shortening fraction (%)at follow-up	44 (IQR 37-47.5)	39 (35-45)	46 (41-48)
Ejection fraction (%)at follow-up	60.5 (IQR 57.5-64.5)	61 (58-65)	60 (56-64)
TR moderate or severe at follow-up	7 (25.0%)	2	5
MR moderate or severe at follow-up	2 (7.1%)	-	2

Data presented as number (percentage) or median (interquartile range).

(Legend: ISHLT: International Society for Heart and Lung Transplantation, eGFR: estimated glomerular filtration rate, TAPSE: tricuspid annular plane systolic excursion, TR: tricuspid regurgitation, MR: mitral regurgitation).

Supplementary legend: Table 1 summarizes the pre-transplant and post-transplant characteristics of ACHD patients requiring HT, overall and among patients requiring additional repair or not.
